# Medicina Translacional e Ciência da Implementação: Como Transformar o que Sabemos no que Efetivamente Fazemos

**DOI:** 10.36660/abc.20211029

**Published:** 2022-07-29

**Authors:** Protásio Lemos da Luz, Francisco Rafael Martins Laurindo

**Affiliations:** 1 Universidade de São Paulo Faculdade de Medicina Instituto do Coração (InCor) São Paulo SP Brasil Instituto do Coração (InCor) da Faculdade de Medicina da Universidade de São Paulo HC-FMUSP, São Paulo, SP – Brasil

**Keywords:** Pesquisa Médica Translacional/tendências, Colesterol, Aterosclerose, Genoma Humano, Inibidores de Hidroximetilglutaril-CoA Redutases/uso terapêutico, Doenças Cardiovasculares/mortalidade, Exercícios, Qualidade de Vida, Medicina Preventiva

A Medicina Translacional engloba basicamente três áreas: 1) a aceleração de transmissão de conhecimentos de pesquisa básica à aplicação clínica; 2) análise causal e da fisiopatologia de observações clínicas, pela interação com ciência básica; e 3) implementação, na população geral, de conhecimentos básicos e conceitos oriundos de pesquisas clínicas e experimentais, também chamada Ciência da Implementação. No passado, muitas descobertas fundamentais ficaram apenas no domínio das ciências básicas, levando-se muitos anos até se transformarem em instrumentos diagnósticos ou tratamentos aplicáveis à prática médica.

Um exemplo elucidativo é a relação entre colesterol e aterosclerose. As primeiras demonstrações de que colesterol induzia aterosclerose foram feitas pelos russos, em coelhos, entre 1908 e 1913.^1^ O Framingham Heart Study,^2^ publicado em 1961, foi o primeiro estudo demostrando esse fato em humanos. No entanto, a primeira estatina só foi produzida em 1976, inaugurando a era atual do tratamento medicamentoso da aterosclerose.^3^ Esse enorme hiato ocorreu em muitas outras circunstâncias e representa um desperdício do conhecimento e de vidas humanas.

## Base da medicina preventiva: estilo de vida sadio

Quando se procura aplicar conhecimentos médicos à população geral, o conceito de estilo de vida sadio deve ser salientado, sobretudo quando se pretende enfatizar a medicina preventiva.

A maioria dos eventos cardiovasculares, tais como infarto do miocárdio e morte, estão associados a fatores de risco como dislipidemia, tabagismo, hipertensão e diabetes.^4^ Fatores genéticos representam a minoria. Outro exemplo é o estudo Whitehall, conduzido na Inglaterra,^5^ que mostrou que trabalhadores públicos que ocupavam posições hierárquicas inferiores no trabalho morriam três a quatro vezes mais que aqueles em posições superiores. A base da medicina preventiva depende de estilo de vida sadio, incluindo dieta predominante em vegetais, frutas e peixes, associada à baixa ingestão de carnes vermelhas e carboidratos. Exercícios aeróbicos e de força, no mínimo 150 minutos/semana são fortemente indicados, inclusive para proteção de funções cognitivas e prevenção de Alzheimer.^6^

Exercícios e dieta são essenciais na prevenção e tratamento do diabetes, hipertensão e obesidade, e vários programas para combate ao tabagismo estão disponíveis, com consideráveis índices de sucesso. No livro Zonas Azuis,^7^ pesquisadores americanos analisaram o estilo de vida das cinco populações mais longevas do mundo: Okinawa (Japão), Sardenha (Itália), Ikaria (Grécia), Loma Linda (Califórnia) e Nicoya (Costa Rica). Alguns costumes são comuns a todos: dieta baseada em grãos, frutas, vegetais e peixes com pouca carne vermelha; vida social intensa; religiosidade; valorização da família; trabalhos braçais rotineiros como andar a pé e cuidar de animais, cozinhar e cuidar da casa; e uso restrito de medicamentos. Fatores genéticos não parecem ser a única explicação dessa longevidade visto que são populações de diferentes países e sem qualquer parentesco.

Estresses emocionais de qualquer origem são fatores causais de eventos cardiovasculares. O aumento exponencial de tais condições durante a pandemia COVID-19 confirmam essas circunstâncias.^8,9^

Um aspecto peculiar do estilo de vida saudável é a dificuldade de implementação em adultos, e isso representa importante desafio para a medicina translacional no seu terceiro componente que é justamente a população geral. Por exemplo, resultados de iniciativas para implementar hábitos saudáveis em crianças e adolescentes, como demonstrado no Brasil e outros países^10,11^ impressionam – as crianças vigiando os pais para que não fumem, façam exercícios e sigam uma boa dieta! Hulsegge et al.,^12^ observaram que indivíduos que mantiveram 4-5 hábitos sadios por cinco anos tiveram 2,5 vezes menos risco de doenças cardiovasculares e mortalidade global comparados aos que não fizeram.

É importante considerar o contexto em que tal implementação ocorre, ou seja, em hospitais, em programas educacionais, no Sistema Único de Saúde (SUS) ou na medicina privada, em consultas *online* e outros. Estratégias diferentes são necessárias dependendo do contexto.

## Medicina de precisão

Hoje prescrevemos medicações com base em resultados de pesquisas que demonstraram que determinadas doses de medicamentos são eficientes. Isso não leva em consideração respostas individuais; ou seja, tratamos a média, sem identificar quem são os respondedores e os não respondedores. Os efeitos colaterais também são relatados assim. Por outro lado, os estudos randomizados não incluem pacientes com comorbidades, e estudam apenas 6-8% da população doente, o que não representa o mundo real. Evidentemente, isso causa erros e dificuldades no ajuste de doses.

A farmacogenética permite uma caracterização mais precisa dos pacientes quanto à resposta a agentes externos, e permitirá uma individualização de tratamentos, como por exemplo, na prevenção de reações alérgicas. Em suma, o conhecimento do genoma humano e das respostas do organismo permitirão a individualização de tratamentos considerando a resposta a contraste, intolerância a agentes externos, e sensibilidade a sal, antiagregantes plaquetários e anticoagulantes. Claro que isso ainda não é prática corrente; mas logo será.

## Desigualdades socioeconômicas tem grande impacto na incidência de doenças

O Whitehall Study^5^ mostrou uma relação entre menor nível de satisfação no emprego e maior mortalidade. Desde então, inúmeros estudos mostraram que o nível educacional, recursos financeiros e níveis sociais influenciam na prevalência de doença e mortalidade;^13^ a causa não é apenas psicológica. Indivíduos melhor posicionados têm mais conhecimento das doenças, têm acesso a melhores centros médicos e podem pagar gastos com saúde. Esse é um problema universal, mais relacionado à economia e desenvolvimento social, mas que se reflete na saúde.

## Comorbidades em idosos e multidisciplinaridade

A população está envelhecendo. Comorbidades como doenças cardiovasculares, cânceres, doenças reumáticas, renais, metabólicas, inflamatórias, urológicas, respiratórias, neurológicas (demências, Alzheimer) e psiquiátricas são muito frequentes entre idosos. Raramente se encontra um paciente idoso com uma só doença. Daí a necessidade de múltiplos especialistas para se tomar a melhor conduta em casos mais complexos.^14,15^ Em consonância com esse conceito, uma metanálise concluiu que um trabalho em equipe (*teamwork*) relaciona-se positivamente com o desempenho clínico.^16^

## Estabelecimento de riscos a médio e longo prazo

Embora escores de risco para doenças cardiovasculares sejam imperfeitos, são muito úteis quando se trata de convencer pacientes em adotar um estilo de vida saudável, em realizar avaliações periódicas e a usar as medicações. Como algumas doenças são silenciosas (hipertensão, diabetes mellitus, aterosclerose) estabelecer riscos é de suma importância prática. Embora os escores mais usados projetam riscos para 10 anos, hoje se calculam riscos em 30 anos.

Técnicas e parâmetros especiais, como o escore de cálcio coronário, radioisótopos e ecocardiograma permitem recalcular riscos, mais precisamente reclassificar indivíduos.^17^ Índices inflamatórios como PCR ultrassensível e escores genômicos também podem contribuir para aperfeiçoar projeção de riscos. Lipoproteínas não convencionais como lipoproteína (a), colesterol não HDL, partículas ricas em triglicéride, apolipoproteína CIII, *angiopoietin-like protein 3* (ANGPTL3), *angiopoietin-like protein* 4 (ANGPTL4), apolipoproteína IV, apolipoproteína E, e variantes gênicas, como PCSK9 podem influenciar no risco cardiovascular.^18^ O grande mérito de calcular riscos é poder argumentar junto aos pacientes sobre a necessidade de vigilância constante e tomada de decisões.

## Uso criterioso de tecnologias: risco versus benefício

Novos avanços tecnológicos são em geral benéficos, porém têm seu lado perigoso. Exemplos são diagnósticos de lesões tireoidianas, mamárias e prostáticas mínimas que desencadearam intervenções “preventivas” desnecessárias.^19^ O mesmo pode-se dizer das técnicas de imagem – cintilografia, tomografias coronárias e intervenções percutâneas – o uso indiscriminado de tais tecnologias sobrecarrega o sistema de saúde, aumenta custos e cria angústia nos pacientes. Países como Reino Unido e Canadá já adotam medidas para evitar “excessos”. No Brasil, também deveríamos adotar medidas para avaliar a qualidade do exercício profissional na medicina (como efetuado, por exemplo, pela Ordem dos Advogados do Brasil). O orçamento federal é insuficiente para atender a maioria da população, que depende do SUS, e não pode admitir desperdiço. Saliente-se aqui a importância dos hospitais de ensino, nos quais técnicas inovadoras podem ser criticamente avaliadas.

## Trabalho em equipe (“*Teamwork*”)

Dada a complexidade de certos casos, comorbidades, diferentes capacidades institucionais e experiências individuais, trabalhar em equipes multiprofissionais é uma maneira eficiente de oferecer o melhor aos pacientes. No caso da cardiologia, normalmente um clínico, um intervencionista, um cirurgião ou arritmologista devem compor a equipe.^20,21^

Na prática, a indicações de procedimentos é influenciada pela experiência individual; por exemplo, hemodinamicistas podem ter preferências por intervenções percutâneas enquanto cirurgiões podem se inclinar por cirurgias. Na verdade, há argumentos que apoiam um ou outro procedimento, baseados no caráter não-invasivo, em registros sobre a evolução a longo prazo bem como na eficiência de tratamentos medicamentosos e estilo de vida do paciente. Além disso, a rápida evolução de técnicas de investigação e tratamentos, e sobretudo a experiência particular dos médicos e centros médicos também contribuem para possíveis diferenças de opiniões. Portanto, o *Heart Team* serve para minimizar esses vieses. Em meio a estas circunstâncias, é preciso lembrar que o paciente deve ser esclarecido e consultado sobre suas preferências.

## Qualidade da pesquisa – base do processo de translação

Os argumentos acima nos levam ao conceito fundamental de que a medicina translacional necessita de alta qualidade científica em todas as suas etapas. Desde a obtenção de dados experimentais *in vitro*, *ex vivo* ou *in vivo* passando por estudos clínicos de fases I a III, até a implementação do conhecimento na população, o rigor científico deve ser observado. Idealmente, estudos clínicos randomizados, com desfechos relevantes e bem definidos, e número de pacientes e tempo de evolução adequados são preferíveis. Uma dificuldade inerente aos estudos randomizados são os altos custos e a demora na obtenção dos resultados. Há fatores que influenciam claramente a implementação de boas práticas à população, como o uso *off label* de medicamentos, influências econômicas, e concepção errônea da aplicação do livre arbítrio médico. Por outro lado, existem hoje técnicas de randomização mendeliana, estudos de associação do genoma completo (*GWAS, Genome Wide Association Studies*), e “big data” com contribuições da inteligência artificial e informática, que possibilitam investigações mais aprofundadas com elucidações de causas e mecanismos fisiopatológicos.^22,23^ No caso de intervenções, a eficiência clínica é o mais importante para o médico. No final, a medicina tem sua credibilidade alicerçada em princípios do método científico.
