# Supplementary material for: Medicina Translacional e Ciência da Implementação: Como Transformar o que Sabemos no que Efetivamente Fazemos
Source: Arq Bras Cardiol. 2022 Jul 29;119(2):342–5. [Article in Portuguese] doi: 10.36660/abc.20211029 (PMC9363070; doi:10.36660/abc.20211029)
Supplement: Translation

# Translational Medicine and Implementation Science: How to Transform What We Know Into What We Do

**Authors:** Protásio Lemos da Luz, Francisco Rafael Martins Laurindo

**Affiliations:** 1 São Paulo SP Brazil Instituto do Coração (InCor) da Faculdade de Medicina da Universidade de São Paulo HC-FMUSP, São Paulo, SP – Brazil

**Keywords:** Translational Medical Research, Cholesterol, Atherosclerosis, Genome Human, Hydroxymethylglutaryl-CoA Reductase Inhibitors/therapeutic use, Cardiovascular Diseases/mortality, Exercises, Quality of Life, Preventive Medicine

Translational Medicine encompasses three areas: 1) acceleration of knowledge transmission from basic science to clinical practice; 2) analysis of the causes and pathophysiology of clinical observations through interaction with basic science; and 3) implementation of basic knowledge and concepts produced by clinical and experimental research in the general population, which is also known as “implementation science”. In the past, some fundamental discoveries stayed confined to the basic science for long years before becoming diagnostic instruments or therapies applicable to practice.

An instructive example is the relationship between cholesterol and atherosclerosis. The first evidence that cholesterol induced atherosclerosis came from studies conducted on rabbits by Russians between 1908 and 1913.^1^ The Framingham Heart Study,^2^ published in 1961, was the first to demonstrate this fact in humans. However, statin was first produced only in 1976, starting the current era of pharmacological treatment of atherosclerosis.^3^ This huge gap occurred in other contexts and represents a waste of knowledge and human lives.

## Basis of preventive medicine: a healthy lifestyle

When medical knowledge is to be applied to the general population, the concept of healthy lifestyle should be highlighted, especially in terms of preventive medicine.

Most cardiovascular events, such as myocardial infarction and death, are associated with risk factors such as dyslipidemia, smoking, hypertension and diabetes.^4^ Genetic factors are less representative. Another example is the Whitehall study, conducted with British civil servants,^5^ that showed that servants in lower grades of employments had a mortality rate three to four times higher than of those in the higher grades. The basis of preventive medicine is related to a healthy lifestyle, including a diet composed predominantly of fruit, vegetable and fish, and low intake of meat and carbohydrates. At least 150 minutes per week of aerobic and strength exercises are strongly recommended, including for protection of cognitive functions and Alzheimer prevention.^6^

Exercise and diet are essential for preventing and treating diabetes, hypertension and obesity, and several anti-smoking programs are currently available, with remarkable success rates. In the book “Blue Zones”,^7^ American researchers evaluated the lifestyle of the five longest-lived communities in the world – Okinawa (Japan), Sardinia (Italy), Ikaria (Greece), Loma Lima (California) and Nicoya (Costa Rica). Some habits were shared by these communities – a diet mainly consisting of grains, fruits, vegetables, and fish, and poor in meat; active social life; religiosity; putting family first; manual labors like walking, taking care of animals, cooking, and taking care of the house; and restricted use of medications. Genetic factors cannot solely explain longevity of these populations, as they live in different countries and have no family relationship.

Emotional stress of any cause is a causal factor of cardiovascular events. The exponential increase of these conditions during the COVID-19 pandemic confirms these circumstances.^8,9^

It is noteworthy, however, that a healthy lifestyle is difficult to be implemented in adults. This represents an important challenge for translational medicine, particularly for its third component that concerns the general population. Results of initiatives to implement healthy habits in children and adolescents, as reported in Brazil and other countries,^10,11^ for example, are impressive – children asking their parents not to smoke, to exercise and to follow a good diet! Hulsegge et al.^12^ found that individuals who maintained four to five healthy lifestyle factors over a five-year period had 2.5 times lower risk of cardiovascular disease and all-cause mortality than those who did not.

In addition, it is important to consider in which context these initiatives have been implemented – in hospitals, educational programs, in the unified health system facilities or in private centers, by online consultations or others. Different contexts require different strategies.

## Precision medicine

Today, medications are prescribed based on results of studies that showed their effective doses, which does not take into account individual responses, *i.e*., doses have been established based on mean responses, without identifying who are responders and non-responders to the treatment. Side effects are described in a similar manner. On the other hand, randomized trials do not include patients with comorbidities and study only 6-8% of patients with the disease, which does not represent the real world. This causes errors and difficulties in adjusting medication doses.

Pharmacogenetics provides a more precise characterization of patients in terms of individualized responses to external agents and may form the basis for tailored therapies, as in preventing allergic reactions. Briefly, the knowledge of the human genome and the body responses will allow individualization of treatments considering the response to contrasts, intolerance to external agents, and sensitivity to salt, antiplatelet agents, and anticoagulants. Although this is not a current practice, it will be soon.

## Socioeconomic inequality has a great impact on disease incidence

The Whitehall study^5^ showed a relationship between a lower level of job satisfaction and higher mortality. Since then, several studies have shown that educational attainment, financial resources and social level have an influence on disease prevalence and mortality^13^ due to factors other than psychological ones. Individuals with higher status are more aware of their diseases, have greater access to better health care and are more able to pay health costs. This is a universal problem that is more related to economy and social development, but affects health.

## Comorbidities in the elderly and Multidisciplinarity

The population is aging. Comorbidities like cardiovascular diseases, cancers, rheumatic, renal, metabolic, inflammatory, urological, respiratory, neurological (dementia, Alzheimer disease) and psychiatric diseases are far more common among the elderly. It is rare to find an older patient with only one disease. For this reason, several specialists would be needed to provide the best care for patients with complex conditions.^14,15^ In fact, a meta-analysis concluded that a teamwork is positively related to clinical performance.^16^

## Establishment of medium and long-term risks

Although cardiovascular risk scores are imperfect, they are helpful in convincing patients to adopt a healthy lifestyle, to undergo periodic evaluations and to comply with medication regimens. Some diseases (e.g. hypertension, diabetes mellitus and atherosclerosis) are “silent” and hence the establishment of risks is of highly practical importance. Although the most used scores estimate 10-year risks, today the risk level of cardiovascular risks is estimated over a 30-year period.

Special techniques and parameters, like the coronary calcium score, radioisotopes and echocardiography allow recalculation of the risk, or more precisely, reclassification of patients.^17^ Inflammatory markers like high-sensitivity C-reactive protein and genomic scores can also improve risk projections. Unconventional lipoproteins may also be helpful, including the lipoprotein(a), non-HDL cholesterol, triglyceride-rich lipoproteins, apolipoprotein CIII, angiopoietin-like protein 3 (ANGPTL3), angiopoietin-like protein 4 (ANGPTL4), apolipoprotein IV, apolipoprotein E, and genetic variants like PCSK9 can influence the cardiovascular risk.^18^ The great advantage of risk calculation is to use it as an instrument to show patients the importance of continuous monitoring and decision making.

## Judicious use of technologies: risks versus benefits

Technological advances are generally beneficial but may be hazardous. For example, the diagnosis of minimal lesions of thyroid, breast and prostate has led to “preventive”, unnecessary interventions.^19^ The same is true for imaging tests – scintigraphy, coronary computed tomography and percutaneous interventions – the indiscriminate use of these technologies overburdens the health care system, increases costs and causes patient anxiety. Countries like the United Kingdom and Canada have adopted measures to prevent “excesses”. In Brazil, the quality of medical practice should be systematically evaluated (as performed by the Order of Attorneys of Brazil). The federal budget is insufficient to cover the health costs of the majority of the population, users of the federal public health system, and thus waste cannot be accepted. In addition, medical school hospitals play an important role in critically evaluating the innovative techniques.

## Teamwork

Due to the complexity of some cases, presence of comorbidities, different institutional capacities and individual experiences, multidisciplinary teams are an effective way to provide the best care to the patients. In Cardiology, multidisciplinary teams should include a clinician, an interventionist, a surgeon and an arrhythmia specialist.^20,21^

In clinical practice, the indication for procedures is influenced by individual experiences. For example, while catheterization specialists may prefer percutaneous interventions, surgeons may be inclined to surgeries. In fact, there are arguments to support one or the other treatment option, based on its non-invasive character, long-term outcomes of the disease, as well as efficacy of previous drug treatments and patient lifestyle. Also, the fast development of assessment tools and therapeutic strategies, and individual experience of physicians and medical centers also contribute to differences of opinions. In this context, the Heart Team serves to minimize these biases. It is also worth emphasizing that the patient should be informed and asked about his/her preferences.

## Research quality – basis of the translational process

The arguments mentioned above lead to the fundamental concept that translational medicine requires high-quality science in its every step. Scientific accuracy must exist from the collection of *in vitro*, *ex vivo* and *in vivo* data, development of phase I and II clinical trials, until the application of knowledge. Ideally, randomized clinical trials, with well-defined, relevant outcomes, and adequate number of patients and time of follow-up are preferred. A difficulty inherent to randomized studies is the high costs and long time to obtain results. There are some factors that have a clear influence on the implementation of good practice in the population, like the off-label use of drugs, economic issues, and an erroneous notion of free will among physicians. On the other hand, methods such as Mendelian randomization, Genome Wide Association Studies (GWAS), and the Big Data, with contributions from artificial intelligence and informatics, allow deeper investigations and elucidation of causes and pathophysiological mechanisms.^22,23^ Regarding interventions, clinical efficacy is the most important issue for physicians. Credibility in Medicine is then grounded in the principles of scientific method.
